# Supplementary material for: GM-Improved Antiaging Effect of Acrylonitrile Butadiene Styrene in Different Thermal Environments
Source: Polymers (Basel). 2019 Dec 28;12(1):46. doi: 10.3390/polym12010046 (PMC7023587; doi:10.3390/polym12010046)
Supplement: Supplementary file 1 [file polymers-12-00046-s001.pdf]

polymers

Supplementary Materials for:

GM-improved anti-aging effect of acrylonitrile-butadiene-styrene in different thermal environments

Yuchao Wang [1,2], Ming Chen [1], Miaoyu Lan [1], Zhi Li [1], Shulai Lu [1*] and Guangfeng wu [2*]

[1] ABS Technology Center of PetroChina, Jilin 132021, China;
[2] School of chemical engineering, Changchun University of Technology, Changchun, China 130012;
* Correspondence: jh_lusl@petrochina.com.cn; gfwu20@163.com;

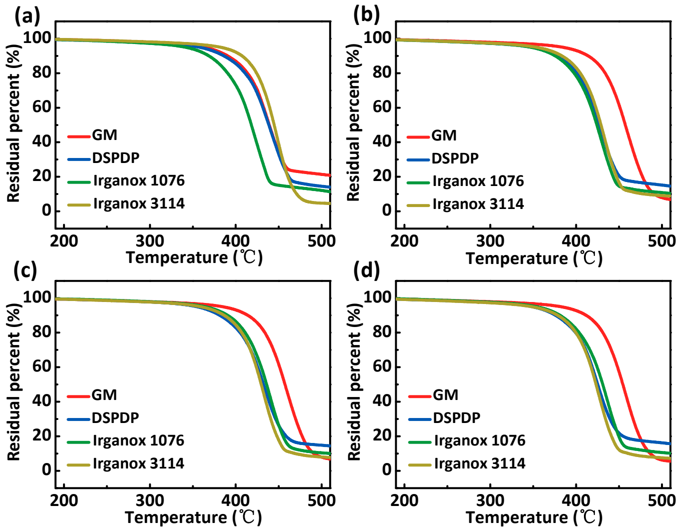

Figure S1. Thermogravimetric analysis (TGA) results for ABS with different additives after different extrusion times: (**a**) the first extrusion; (**b**) the third extrusion; (**c**) the fifth extrusion; (**d**) the sixth extrusion.

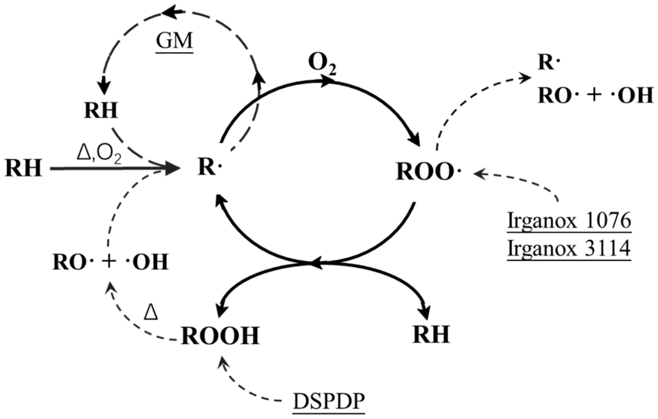

Figure S2. General scheme of thermal-oxidative degradation of ABS resin and its inhibition mechanism by GM, DSPDP, Irganox 1076, and Irganox 3114.

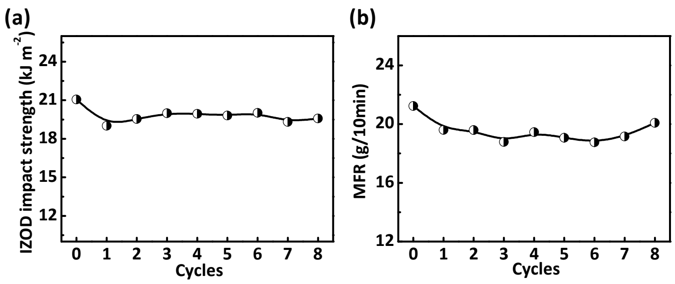

Figure S3. (**a**) Notched impact strength of pure ABS aged by thermal oven. (**b**) Melt flow rate of pure ABS aged by thermal oven.

© 2020 by the authors. Submitted for possible open access publication under the terms and conditions of the Creative Commons Attribution (CC BY) license (http://creativecommons.org/licenses/by/4.0/).
